# Global trends and hotspots in robotic-assisted arthroplasty from 1992 to 2025: a bibliometric and visualized analysis

**DOI:** 10.1051/sicotj/2026012

**Published:** 2026-05-19

**Authors:** Yuanzhuo Xu, Shengdong Yang, Fuqiang Gao, Sébastien Lustig, Weiguo Wang

**Affiliations:** 1 Department of Orthopedics, China-Japan Friendship Hospital (Institute of Clinical Medical Sciences), Chinese Academy of Medical Sciences & Peking Union Medical College Beijing 100029 PR China; 2 Department of Orthopedic Surgery and Sport Medicine, FIFA Medical Center of Excellence, Croix-Rousse Hospital, Lyon University Hospital Lyon PR France; 3 Center for Hip Preservation, Osteonecrosis and Developmental Dysplasia of the Hip, China-Japan Friendship Hospital Beijing 100029 PR China; 4 Department of Orthopedics, China-Japan Friendship Hospital Beijing 100029 PR China

**Keywords:** Robotic surgical procedure, Arthroplasty, Bibliometrics, VOSviewer, CiteSpace

## Abstract

*Introduction*: Robotic-assisted arthroplasty (RAA) is a rapidly advancing technology in orthopedic surgery. This study aims to conduct a comprehensive bibliometric analysis to map the global research landscape, identify hotspots, and trace the evolution of this field from 1992 to 2025. *Methods*: Literature was retrieved from the Science Citation Index Expanded (SCI-E) and Social Sciences Citation Index (SSCI) of Web of Science Core Collection. CiteSpace (6.3.R1) and VOSviewer (1.6.20) were employed to perform quantitative and visualized analyses of countries, institutions, authors, journals, co-citations, and keywords. *Results*: A total of 1373 publications were included. The annual publication output demonstrated exponential growth, particularly in the last five years. The USA led in productivity, while the United Kingdom had the highest average citation frequency. Mont, Michael A., Batailler, Cécile, and Lustig, Sébastien were identified as the most prolific authors. *The Journal of Arthroplasty* published the most papers. Keyword analysis revealed that research hotspots focused on total knee arthroplasty, alignment, accuracy, and functional alignment. Bursts and timeline analyses indicated a shift in frontiers from alignment accuracy and clinical outcomes to updates of robotic systems and new technologies. *Conclusion*: This bibliometric analysis maps the evolution of RAA, highlighting a shift from technical precision to patient-centered outcomes, and identifies future directions, including long-term benefit assessment and technology integration.

## Introduction

Osteoarthritis (OA) is a prevalent degenerative joint disease and a leading cause of pain and disability worldwide [1]. Total joint arthroplasty, particularly of the hip and knee, has been established as a highly successful and cost-effective intervention for end-stage OA, significantly improving patients' quality of life [2]. However, conventional manual techniques are susceptible to variability in surgical precision, which can affect prosthetic alignment, soft-tissue balance, and ultimately, clinical outcomes and implant longevity [3].

The development of robotic-assisted (RA) systems promotes innovation in orthopedic surgery, aiming to enhance the accuracy and consistency of arthroplasty procedures. Over the past two decades, the use of robotics in arthroplasty has shifted from an experimental approach to an established clinical option, supported by a growing body of research evaluating its effectiveness, safety, and clinical outcomes [4, 5]. However, large-scale bibliometric analysis is lacking. Therefore, this study employs CiteSpace and VOSviewer, two powerful scientific visualization tools, to conduct a bibliometric and visualized analysis of the literature on robotic-assisted arthroplasty (RAA) from 1992 to 2025. This study emphasizes the conceptual evolution (e.g., mechanical to functional alignment (FA), sensor integration, and system-level innovation) as a central analytical focus, aiming to enable researchers and clinicians to more intuitively grasp the hotspots and evolving trends in this field.

## Materials and methods

### Literature retrieval and screening

**Figure 1:**
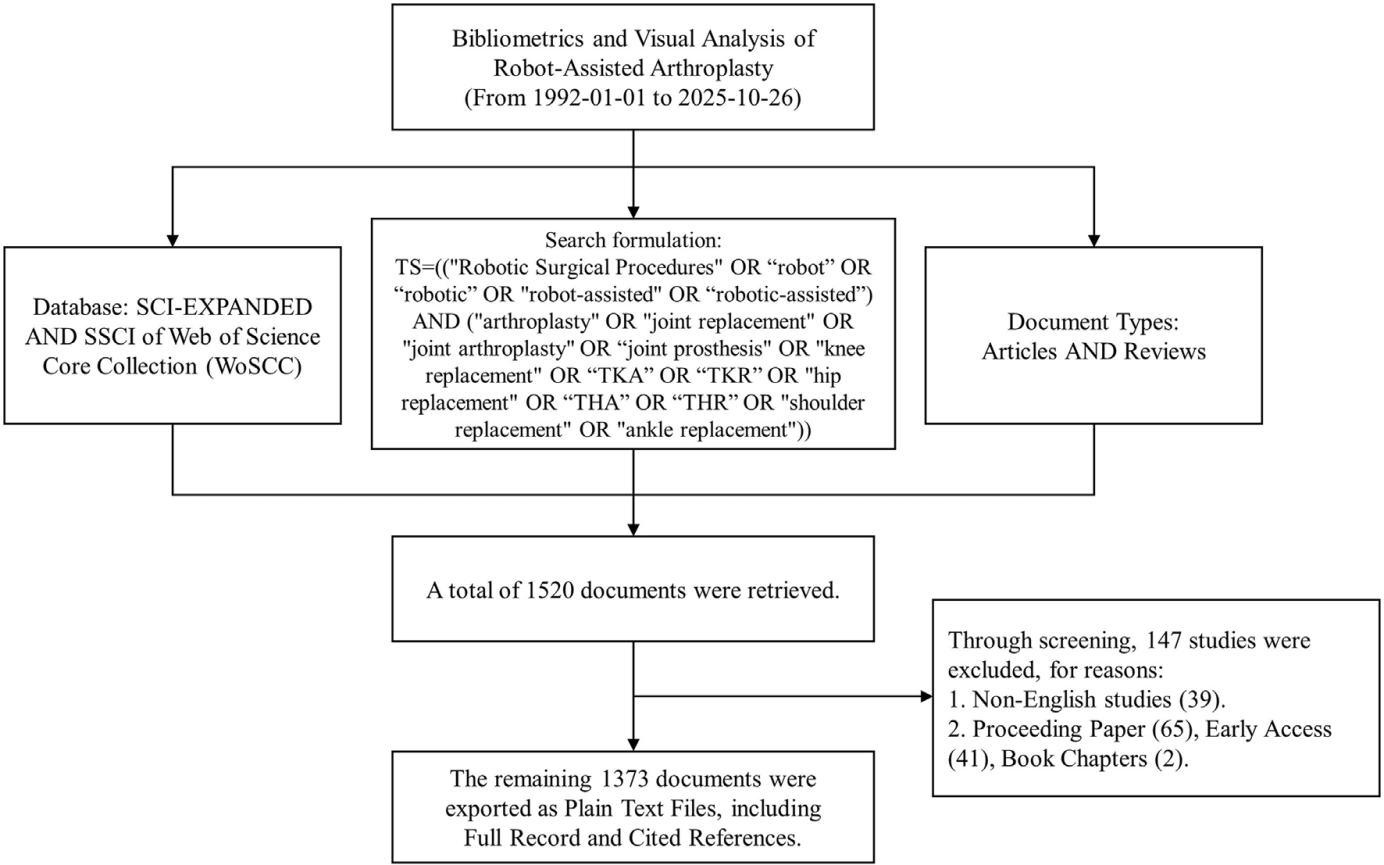
The literature search and screening flowchart.

The Science Citation Index Expanded (SCI-E) and Social Sciences Citation Index (SSCI) of Web of Science Core Collection (WoSCC) served as the data source due to their data availability, interdisciplinary coverage, and academic authority [6]. The study was conducted using the “topic search” method, which searches the title, abstract, and indexing. Query: TS=((“Robotic Surgical Procedures” OR “robot” OR “robotic” OR “robot-assisted” OR “robotic-assisted”) AND (“arthroplasty” OR “joint replacement” OR “joint arthroplasty” OR “joint prosthesis” OR “knee replacement” OR “TKA” OR “TKR” OR “hip replacement” OR “THA” OR “THR” OR “shoulder replacement” OR “ankle replacement”));

The inclusion and exclusion criteria were as follows: Inclusion criteria: (1) Directly obtained through the search query; (2) Database: SCI-E and SSCI; (3) Publication date: From 1992-01-01 to 2025-10-26; (4) Document types: Articles and Reviews, because they often represent the presentation and summary of creative achievements, they best reflect the cutting-edge progress in a particular field; (5) Language: English. Exclusion criteria: (1) Non-English studies; (2) Proceeding Papers, Early Access, Book Chapters. Two independent reviewers performed literature retrieval and screening; disagreements were resolved by consensus. Eventually, 1373 records were exported as plain text, including the full record and cited references. Then duplicates were removed through CiteSpace. Figure 1 illustrates the workflow of the screening process.

### Bibliometrics

Bibliometrics is a quantitative analysis method for the collection, analysis, and description of published literature [7]. Its content primarily includes simple literature counts, such as keyword frequency, cited literature, and basic quantitative information regarding publications, authors, journals, countries, and institutions [8].

### Research tools

This study used CiteSpace (version 6.3.R1) and VOSviewer (version 1.6.20) to draw the visual maps. CiteSpace can generate clustering maps and timeline maps of keywords that allow us to understand the trends and clusters of keywords over time. VOSviewer can draw visual maps of keywords, co-authors, and collaborating institutions.

## Results

### Basic quantitative information

**Figure 2:**
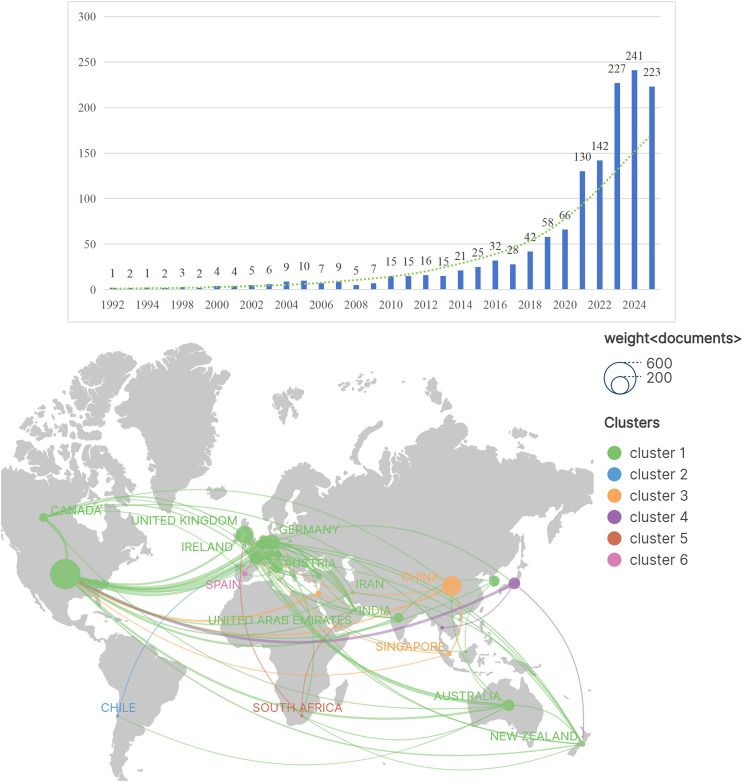
A: Trends in the number of publications in the field of robot-assisted arthroplasty from 1992-01-01 to 2025-10-26. B: Geographic distribution map of the top 31 countries with the largest number of publications.

A total of 1373 publications, involving 5256 authors from 1696 institutions across 54 countries, were published in 206 journals and received 23775 citations from 4989 sources. The annual number of published articles has shown a marked increase, rising from ≤10 (1992–2009) to over 100 (2021 onward), with particularly rapid growth in the past five years (Figure 2A). The relatively low count for 2025 is attributable to the inclusion of literature only from its first ten months. This overall trend indicates significant progress and strong future potential for the field. A total of 54 countries have contributed publications on RAA (Figure 2B).

Mont, Michael A. (37 articles), Batailler, Cécile (37), and Lustig, Sébastien (36) are the most prolific authors from 1992 to 2025 (Table 1). Mont, Michael A., also holds the highest average citation frequency (46.5 citations/article), establishing him as a leading and influential scholar from the Cleveland Clinic Foundation, known for clinical studies on hip and knee replacement outcomes. His sustained influence is further evidenced by several landmark publications, such as a comprehensive review of RA total knee arthroplasty (TKA) [9] and a critical appraisal of contemporary robotic platforms [10], both of which have been widely cited for synthesizing evidence and guiding clinical decision-making.

**Table 1:** The top five authors and countries in the field of robotic-assisted arthroplasty.

Rank	Author	Documents	Average citation frequency
Authors			
1	Mont, Michael A.	37	46.5
2	Batailler, Cécile	37	27.2
3	Lustig, Sébastien	36	25.4
4	Servien, Elvire	29	27.5
5	Pearle, Andrew D.	27	33.5
Countries			
1	USA	486	26.2
2	CHINA	199	9
3	UNITED KINGDOM	166	35
4	GERMANY	119	20.3
5	ITALY	85	15

Batailler, Cécile, and Lustig, Sébastien, as part of the same research group at Lyon University Hospital, have made substantial contributions to the field through systematic evaluations and technical descriptions of contemporary robotic systems. Their 2021 systematic review of the MAKO CT-based robotic system synthesized evidence from 26 studies, demonstrating that robotic-assisted TKA reduces postoperative pain, improves implant positioning accuracy, and achieves equivalent or superior one-year functional outcomes compared to conventional techniques [11]. They also provided a detailed technical description of the ROSA Knee system, elucidating its collaborative surgical workflow and potential for personalized TKA through both image-based and imageless approaches [12]. In a broader context, their comprehensive review of new technologies in knee arthroplasty integrated current concepts, including patient-specific instrumentation, customized implants, sensors, accelerometers, and robotic assistance, offering a critical appraisal of their advantages and limitations [13]. Collectively, these works have guided surgeons in understanding and adopting emerging technologies in knee arthroplasty.

**Figure 3:**
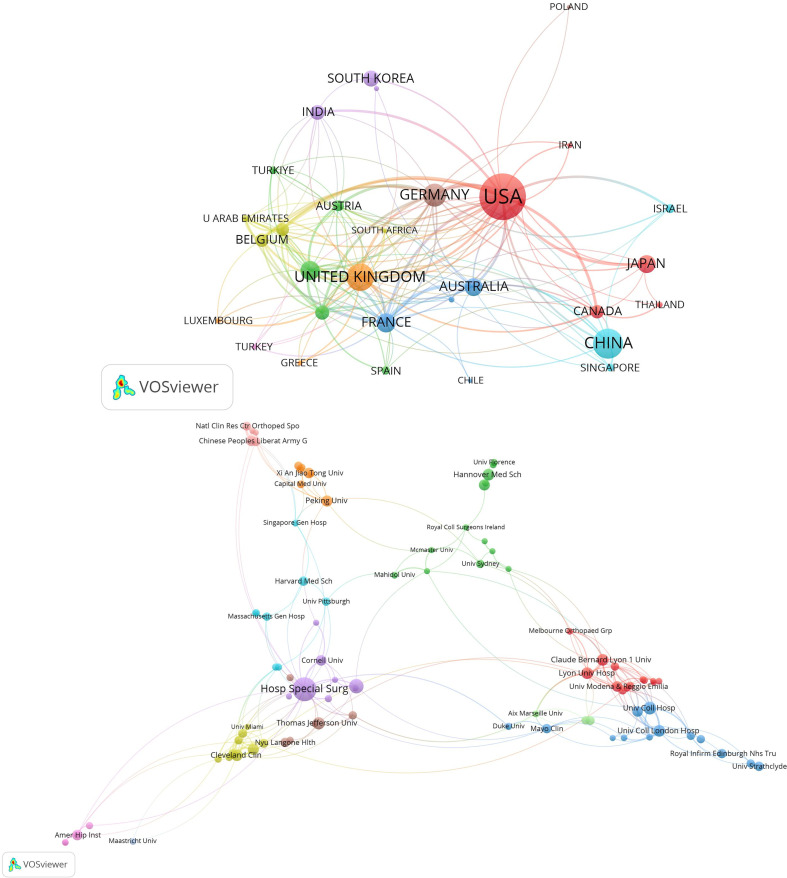
A: Co-occurrence map of countries in terms of the number of publications. B: Co-occurrence map of institutions in terms of the number of publications.

Analysis of the top 31 countries reveals distinct collaborative networks (Figure 3A). While the USA dominates in productivity (486 articles), the UK leads in average citation frequency (35 citations/article) (Table 1). The dominance of the USA in publication output can be attributed to its robust research funding, early adoption of robotic technology in high-volume clinical settings, and effective public-sector support for translating engineering innovations into surgical practice [14]. China, despite high output, receives relatively less attention per article, suggesting its research may face significant limitations that need to be addressed.

*The Journal of Arthroplasty* (130 articles), *Knee Surgery, Sports Traumatology, Arthroscopy* (122), and *Journal of Robotic Surgery* (68) are the most productive journals. Despite a modest publication volume, the *Bone & Joint Journal* has the highest average citation frequency (49.8), confirming its status as a core and highly influential journal in the field. These publications serve as key sources of cutting-edge and high-quality clinical research in RAA.

Institutional analysis highlights that most high-contributing institutions are located in Europe and America (Figure 3B). The Hospital for Special Surgery (84 articles), Cleveland Clinic (52), and University College London (45) are top contributors. Connections between collaborative groups appear less close. Strengthening these inter-group collaborations is important to mitigate potential research bias from factors such as racial differences.

### Keyword analysis

#### Keyword co-occurrence

**Figure 4:**
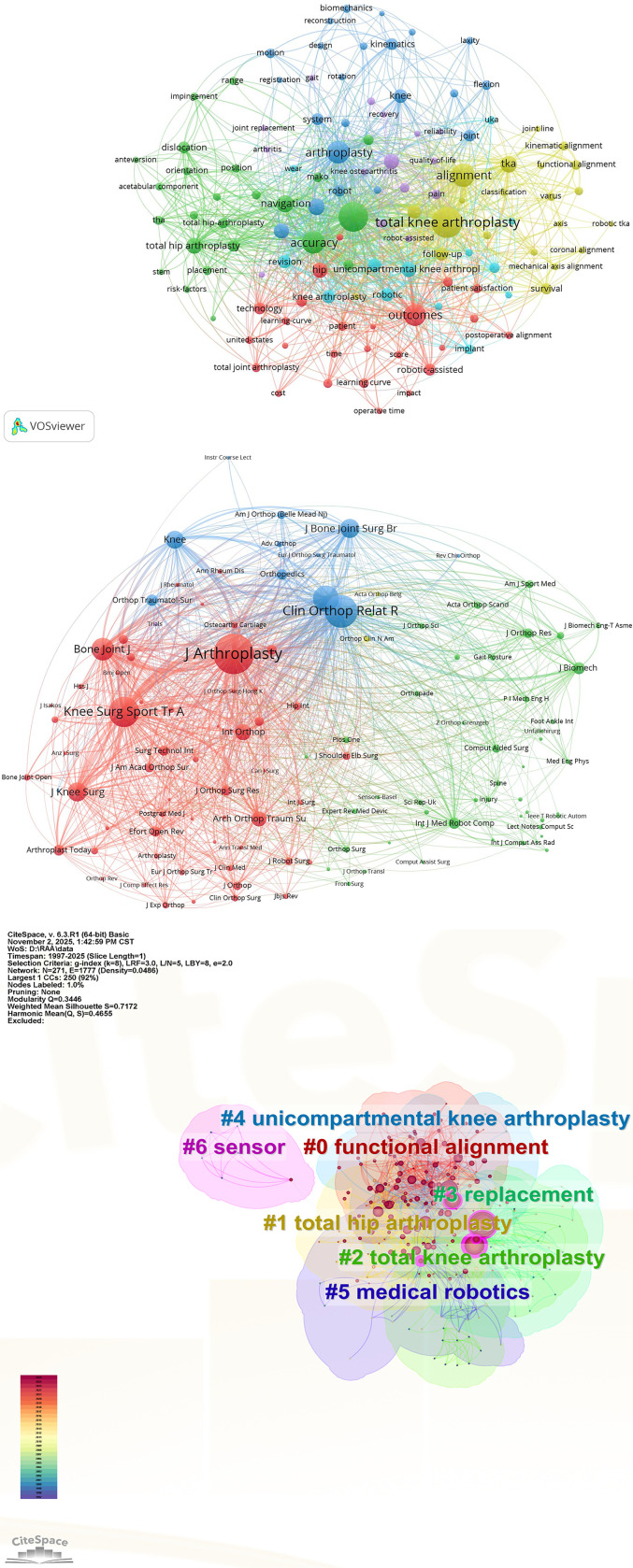
A: Keyword co-occurrence network. B: Journal co-citation network. C: Keyword clustering map. Some cluster labels were refined in the text, such as #3 Clinical outcomes & implant performance and #5 Robotic system evolution.

The keyword co-occurrence network (Figure 4A) identified 120 high-frequency keywords (frequency ≥16). The top five keywords by frequency were “total knee arthroplasty” (433), “replacement” (381), “alignment” (220), “accuracy” (187), and “outcome” (179). By centrality, the top keywords were “replacement” (0.24), “surgery” (0.22), “TKA” (0.19), “alignment” (0.12), and “arthroplasty” (0.12) (Table 2). This underscores “alignment” as a central and enduring focus in RAA research.

**Table 2:** The top five keywords in frequency and centrality in the field of robotic-assisted arthroplasty.

Rank	Count	Keyword	Centrality	Keyword
1	433	Total knee arthroplasty	0.24	Replacement
2	381	Replacement	0.22	Surgery
3	220	Alignment	0.19	Total knee arthroplasty
4	187	Accuracy	0.12	Alignment
5	179	Outcome	0.12	Arthroplasty

#### Keyword clustering

Then, the keyword clustering analysis was conducted by the log-likelihood ratio (LLR) algorithm in CiteSpace. In this study, the Modularity Q was 0.3446, and the Weighted Mean Silhouette S was 0.7172, both exceeding the commonly accepted thresholds (Q > 0.3, S > 0.7). These values indicate robust structural validity and high internal coherence of the clustering results. Clustering analysis revealed 7 major research themes (Figure 4C). To improve interpretability, we manually refined them based on the core publications within each cluster. Among them, #0 functional alignment is a gradually popularizing alignment concept used in TKA. #1 Total hip arthroplasty (THA) is the second major application of robotic assistance; #2 TKA constitutes the dominant surgical focus of the field; *#3 Clinical outcomes & implant performance* encompasses studies on postoperative function, implant survivorship, and complications; #4 Unicompartmental knee arthroplasty (UKA) is a relatively novel but expanding indication for robotics; *#5 Robotic system evolution* covers technological advancements, system development, system-specific learning curves, and workflow integration; and #6 Sensor refers to intraoperative tools for soft-tissue balancing and gap assessment.

#### Keyword bursts and timeline

**Figure 5:**
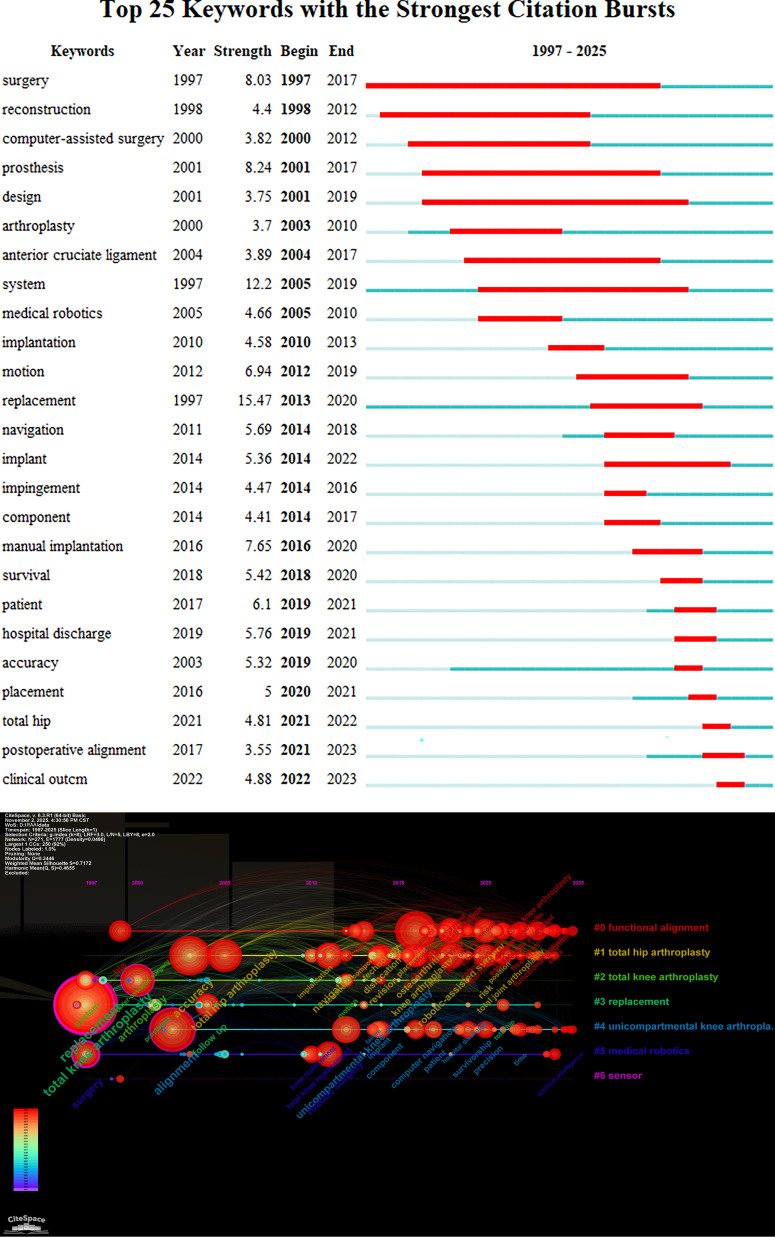
A: Top 25 keywords with the strongest citation bursts from 1997 to 2025. The red line indicates the duration of keyword bursts. B: Keyword evolution timeline from 1992 to 2025. Different colors indicated different clusters. Each circle represents a keyword, corresponding to its respective year in sequence and arranged chronologically. The curves between circles represent the correlation between keywords. Some cluster labels were refined in the text, such as #3 Clinical outcomes & implant performance and #5 Robotic system evolution.

The keyword bursts analysis identified 25 keywords with the strongest citation surges (Figure 5A). Aligning these bursts with their temporal occurrence reveals distinct, technology-driven shifts in research foci. From 1997 to 2005, early bursts of surgery, reconstruction, and computer-assisted surgery coincided with the maturation of conventional arthroplasty and the initial exploration of computer navigation. Between 2005 and 2014, surges in system, medical robotics, and navigation reflected clinical adoption of dedicated robotic platforms (e.g., MAKO, ROSA), while subsequent bursts of motion and implantation signalled growing interest in postoperative motion range of joints and implant positioning accuracy. During 2014–2019, the strongest bursts of implant, survival, and manual implantation marked the peak of comparative effectiveness research contrasting robotic versus conventional techniques. From 2019 to 2025, bursts of accuracy, patient, hospital discharge, postoperative alignment, and clinical outcome signify a shift from technical precision to patient-reported outcome measures (PROMs). This temporal mapping demonstrates how technological breakthroughs and evolving surgical philosophies have sequentially shaped the RAA research agenda.

The timeline visualization (Figure 5B) further corroborates these shifts. #1 THA and #4 UKA, with their long-standing presence, reflect the enduring relevance of hip and knee arthroplasty. However, the rapid expansion of #0 Functional Alignment after 2015 and the resurgence of #5 Robotic system evolution in the last five years are directly attributable to the integration of robotic platforms with individualized alignment targets.

### Co-citation analysis

As shown in Figure 4B, the journal co-citation network primarily consists of three clusters, corresponding to the colors red, green, and blue. The top three cited journals were *The Journal of Arthroplasty* (6252 citations), *Clinical Orthopaedics and Related Research* (4102 citations), and *Knee Surgery, Sports Traumatology, Arthroscopy (KSSTA)* (3501 citations). These are all medical journals with strong influence, focusing on joint replacement, basic biological, and engineering research on orthopedic diseases. This study further analyzed the co-citation of the literature. Using VOSviewer analysis, the top five references cited in this field from 1992 to 2025 are listed in the following table (Table 3). The first article suggested that RA-TKA may enhance the accuracy of mechanical axis alignment outliers and improve the ability to achieve flexion-extension gap balance, without differences in clinical outcomes compared to conventional manual techniques [15]. However, the second and third articles indicate that, compared with conventional jig-assisted TKA, RA-TKA not only improves the accuracy of prosthesis positioning and limb alignment but also reduces pain, facilitates early functional recovery, and shortens the time required for patient discharge [16, 17]. Therefore, while the benefits of RA-TKA for prosthesis positioning, lower limb alignment, and gap balancing are well-established, its impact on clinical outcomes remains controversial.

**Table 3:** The top five co-cited references in the field of robotic-assisted arthroplasty.

Rank	Cited reference	Citations
1	Robotic-assisted TKA Reduces Postoperative Alignment Outliers and Improves Gap Balance Compared to Conventional TKA. doi 10.1007/s11999-012-2407-3 [15]	172
2	Robotic-arm assisted total knee arthroplasty is associated with improved early functional recovery and reduced time to hospital discharge compared with conventional jig-based total knee arthroplasty: a prospective cohort study. doi 10.1302/0301-620X.100B7.BJJ-2017-1449.R1 [16]	159
3	Robotic-arm assisted total knee arthroplasty has a learning curve of seven cases for integration into the surgical workflow but no learning curve effect for accuracy of implant positioning. doi 10.1007/s00167-018-5138-5 [17]	149
4	Robotics in Arthroplasty: A Comprehensive Review. doi 10.1016/j.arth.2016.05.026 [18]	147
5	Robotic-Arm Assisted Total Knee Arthroplasty Demonstrated Greater Accuracy and Precision to Plan Compared with Manual Techniques. doi 10.1055/s-0038-1641729 [19]	137

## Discussion

This study presents a comprehensive bibliometric analysis of the global research landscape in RAA over the past three decades and demonstrates the intellectual structure, collaborative networks, and dynamic evolution of this rapidly advancing field. The research related to RAA has been growing rapidly in recent years, particularly during the periods of 2020–2021 and 2022–2023. This trend reflects the strong interest and confidence that the orthopedic community holds in robotic technology. Our analysis identified Mont, Michael A., Batailler, Cécile, and Lustig, Sébastien as the most prolific authors, with Mont also being the most influential. Institutions such as the Hospital for Special Surgery and the Cleveland Clinic are identified as the core of research productivity. The visualizations reveal distinct, closely connected collaborative groups, primarily within North America and Europe. The lower average citation frequency of high-output countries like China highlights a geographical imbalance in research efforts. Enhancing international collaboration, especially across continents, is crucial for reducing regional bias (e.g., in prosthesis design for different ethnic anatomies) and accelerating global knowledge transfer.

### Intellectual structure and thematic evolution

In terms of keywords co-occurrence and clustering, “arthroplasty”, “replacement”, “alignment”, “accuracy”, and “outcome” spring up, indicating that the accuracy of lower limb alignment during joint replacement surgery and the clinical outcomes of patients are the most focused areas in this field. However, bibliometric prominence does not automatically equate to conceptual innovation. While “alignment” has been a common keyword for decades, its re-emergence within the distinct cluster #0 functional alignment signals a genuine paradigm shift – from a rigid mechanical alignment (MA) philosophy toward personalized, patient-specific targets. This transition is supported by robotic systems capable of executing individualized plans [15, 20]. Similarly, the formation of clusters #5 Robotic system evolution and #6 Sensor reflects a broadening of research focus beyond implant positioning to encompass system design, workflow integration, and intraoperative soft-tissue assessment.

### Bursts analysis and frontier dynamics

The bursts analysis indicates how research priorities have evolved in response to technological milestones. It corroborates the thematic shifts observed in clustering, with an early focus on navigation and systems, followed by comparative effectiveness research, and recently a pivot toward PROMs. This temporal mapping demonstrates that the RAA research agenda has been sequentially shaped by technology availability, comparative validation, and now outcome optimization.

### Mapping bibliometric trends to clinical practice

For the practicing orthopaedic surgeon, the hotspots identified in this analysis closely mirror the real-world application scenarios of robotic systems. In TKA, the sustained dominance of “alignment” and “accuracy” reflects the widespread clinical emphasis on precise component positioning. The rapid ascent of “functional alignment” since 2018 marks its growing acceptance as a viable alternative to MA. In THA, the comparatively lower but steady volume of robotic literature corresponds to the later entry of robotic platforms into hip surgery and the ongoing debate regarding their benefits. In UKA, the expanding cluster (#4) echoes that robotics may be particularly valuable in this technically demanding, less forgiving procedure.

### Research gaps and ongoing controversies

As highlighted in the co-citation analysis, the debate on whether RAA improves clinical outcomes persists [9–11]. Moreover, evidence on RA-THA [4, 5] and UKA [21] remains relatively scarce, and the added value of sensor-guided balancing [22] has yet to be confirmed in large-scale, long-term trials. These gaps are not merely academic – they directly influence surgical decision-making, hospital investment, and reimbursement policy.

Based on our analysis, several promising future research directions emerge: There is a pressing need for long-term and randomized controlled trials focusing on PROMs, implant survivorship, and cost-effectiveness of RAA versus conventional arthroplasty. Robot learning integration can analyze large datasets from pre-operative imaging and intraoperative sensors, enabling predictive planning and autonomous adjustments for optimal balance and alignment.

### Limitations

This study has several limitations. Firstly, the data were sourced exclusively from WoSCC, potentially overlooking relevant studies published in other databases (such as PubMed and Scopus) or in non-English journals, particularly for clinically oriented research. Secondly, the query is broad and may retrieve robotic-related studies not directly relevant to arthroplasty. These studies are difficult to sort out merely due to the limitations of document types and language. Third, the data for 2025 only covers approximately the first ten months, which may underestimate the number of publications in 2025 and influence trend interpretation. Fourth, “influence” is interpreted in a bibliometric sense, not necessarily reflecting clinical superiority or adoption. Fifth, the analysis primarily focuses on quantitative metrics (such as publication volume and citation counts), making it difficult to distinguish the research quality. Sixth, due to the time limit of CiteSpace (version 6.3.R1), keyword clustering, bursts and timeline analyses only examined the trends from 1997 to 2025. However, this limitation will not significantly affect the analysis of hotspots and trends. Finally, this study is subject to time-lag bias, a common limitation in citation-based bibliometric analyses. The citation count and average citation frequency are time-dependent, naturally favoring older publications, which may underrepresent the impact of recent creative work.

## Conclusions

This analysis reveals the emergence of functional alignment, sensor integration, and robotic system evolution as distinct research clusters. Future research should prioritize long-term randomized clinical trials on PROMs and survivorship, while exploring AI integration.

## Abbreviations

FA: functional alignment
MA: mechanical alignment
OA: osteoarthritis
PROMs: patient-reported outcome measures
RA: robotic-assisted
RAA: robotic-assisted arthroplasty
THA: total hip arthroplasty
TKA: total knee arthroplasty
WoSCC: Web of Science Core Collection
UKA: unicompartmental knee arthroplasty

## Data Availability

This article has no associated data generated.
